# Multisite NHERF1 phosphorylation controls GRK6A regulation of hormone-sensitive phosphate transport

**DOI:** 10.1016/j.jbc.2021.100473

**Published:** 2021-02-24

**Authors:** Maria Vistrup-Parry, W. Bruce Sneddon, Sofie Bach, Kristian Strømgaard, Peter A. Friedman, Tatyana Mamonova

**Affiliations:** 1 Center for Biopharmaceuticals, Department of Drug Design and Pharmacology, University of Copenhagen, Copenhagen, Denmark; 2 Laboratory for GPCR Biology, Department of Pharmacology and Chemical Biology, University of Pittsburgh School of Medicine, Pittsburgh, Pennsylvania, USA

**Keywords:** PDZ domain, parathyroid hormone (PTH), G protein–coupled receptor kinase 6A (GRK6A), phosphate transport, PDZ-ligand interaction, binding affinity, simulation, CFTR, cystic fibrosis transmembrane conductance regulator, C-terminal, carboxy-terminal, EBD, ezrin-binding domain, FA, fluorescence anisotropy, GRK6A, G protein–coupled receptor kinase 6A, GRK6Act-22, -^553^QRLFSRQDCCGNCSEEELPTRL^576^, GRK6Act-9, -^568^SEEELPTRL^576^, NHERF1, Na^+^/H^+^-exchanger regulatory factor-1, NPT2A, type II sodium-dependent phosphate cotransporter, OK cells, opossum kidney cells, OKH, opossum kidney clone H, pSer^162^, phosphorylated Ser^162^, PTH, parathyroid hormone, TBST, Tris-buffered saline plus Tween 20

## Abstract

The type II sodium-dependent phosphate cotransporter (NPT2A) mediates renal phosphate uptake. The NPT2A is regulated by parathyroid hormone (PTH) and fibroblast growth factor 23, which requires Na^+^/H^+^ exchange regulatory factor-1 (NHERF1), a multidomain PDZ-containing phosphoprotein. Phosphocycling controls the association between NHERF1 and the NPT2A. Here, we characterize the critical involvement of G protein–coupled receptor kinase 6A (GRK6A) in mediating PTH-sensitive phosphate transport by targeted phosphorylation coupled with NHERF1 conformational rearrangement, which in turn allows phosphorylation at a secondary site. GRK6A, through its carboxy-terminal PDZ recognition motif, binds NHERF1 PDZ1 with greater affinity than PDZ2. However, the association between NHERF1 PDZ2 and GRK6A is necessary for PTH action. Ser^162^, a PKCα phosphorylation site in PDZ2, regulates the binding affinity between PDZ2 and GRK6A. Substitution of Ser^162^ with alanine (S^162^A) blocks the PTH action but does not disrupt the interaction between NHERF1 and the NPT2A. Replacement of Ser^162^ with aspartic acid (S^162^D) abrogates the interaction between NHERF1 and the NPT2A and concurrently PTH action. We used amber codon suppression to generate a phosphorylated Ser^162^(pSer^162^)-PDZ2 variant. *K*_D_ values determined by fluorescence anisotropy indicate that incorporation of pSer^162^ increased the binding affinity to the carboxy terminus of GRK6A 2-fold compared with WT PDZ2. Molecular dynamics simulations predict formation of an electrostatic network between pSer^162^ and Asp^183^ of PDZ2 and Arg at position −1 of the GRK6A PDZ-binding motif. Our results suggest that PDZ2 plays a regulatory role in PTH-sensitive NPT2A-mediated phosphate transport and phosphorylation of Ser^162^ in PDZ2 modulates the interaction with GRK6A.

Na^+^/H^+^ exchange regulatory factor-1, NHERF1 (*SLC9A3R1*), also known as the ezrin-binding phosphoprotein of 50 kDa, belongs to the NHERF family (1) and is the only known PDZ-containing scaffold that controls protein localization at the apical plasma membrane of polarized epithelial cells (2, 3). NHERF1 tethers potential binding partners through tandem PDZ domains named for the common structural domain shared by the postsynaptic density protein, *Drosophila* disc large tumor suppressor, and zonula occludens-1 protein, and the carboxy-terminal (C-terminal) ezrin-binding domain (EBD) associated with ezrin (4). The association between NHERF1 and the type II sodium-dependent phosphate cotransporter (NPT2A) (*SLC34A1*), the primary renal Na-dependent phosphate transporter, is required for hormone-regulated phosphate transport mediated by the NPT2A (4). Loss-of-function mutations in NHERF1 or the NPT2A disrupt phosphate metabolism and lead to hypophosphatemia (5, 6, 7). Parathyroid hormone (PTH) and fibroblast growth factor 23 downregulate the NPT2A–NHERF1 binary complex by activating two distinct signaling pathways that converge at NHERF1 (8, 9, 10, 11).

NHERF1 PDZ1 and PDZ2 domains have similar sequences and identical core-binding motifs (-GYGF-) essential for PDZ-ligand interactions (12, 13). NHERF1 PDZ1 and PDZ2 bind the target-sequence of ligand partners through a short C-terminal linear interaction fragment that is three to four residues in length (X-S/T-X-Φ_COO_^−^ class I PDZ-recognition motifs, where X is any amino acid and Φ is a hydrophobic residue) (14). By convention, these residues are numbered starting from the last position (P^0^) and going backward to P^−1^, P^−2^, P^−3^, and so forth.

Prior experimental studies revealed that G protein–coupled receptor kinase 6A (GRK6A) possessing a canonical PDZ ligand motif (-TRL^576^) at its C terminus binds NHERF1 PDZ domains (15). Most binding is associated with PDZ1. However, a minor interaction between GRK6A and the PDZ2 domain is critical for constitutive or PTH-induced phosphorylation of NHERF1 at Ser^290^ (15, 16) and for PTH-sensitive phosphate transport (16). The biological puzzle is how does PDZ2 become accessible to GRK6A and what is the role of PTH in this process. Solving this dilemma is the goal of this study. We hypothesized that enzymatic phosphorylation of Ser^162^ located adjacent to the PDZ2 core-binding motif (-^163^GYGF^166^-) and incorporation of a double-charged phosphate group promote association between PDZ2 and GRK6A. Ser^162^ is a defined PKCɑ phosphorylation site (17, 18). Notably, PKC is a major pathway of PTH signaling (19, 20, 21) and is the only PKC isoform with a PDZ-recognition motif at its C terminus (-SAV^672^) (22, 23). It remains to be established whether the interaction between NHERF1 and PKCα is necessary for phosphorylation of Ser^162^.

The experiments described here use a variety of complementary approaches to characterize the role of PDZ2 with phosphodeficient Ser^162^ on NHERF1-dependent PTH-sensitive phosphate uptake. Binding determinants of the interaction between PDZ2 or PDZ2 with phospho-Ser^162^, introduced genetically in NHERF1 using amber codon suppression, for the first time, and GRK6A were evaluated using fluorescence anisotropy (FA) and confirmed by molecular dynamics (MD) simulations. Our preliminary study suggests that a double-charged phospho-Ser^162^ is a key determinant of PTH signaling and is required for NHERF1-dependent NPT2A-mediated PTH-sensitive phosphate uptake.

## Results

### Direct interaction between NHERF1 and GRK6A is essential for NPT2A-mediated PTH-sensitive phosphate transport

**Figure 1:**
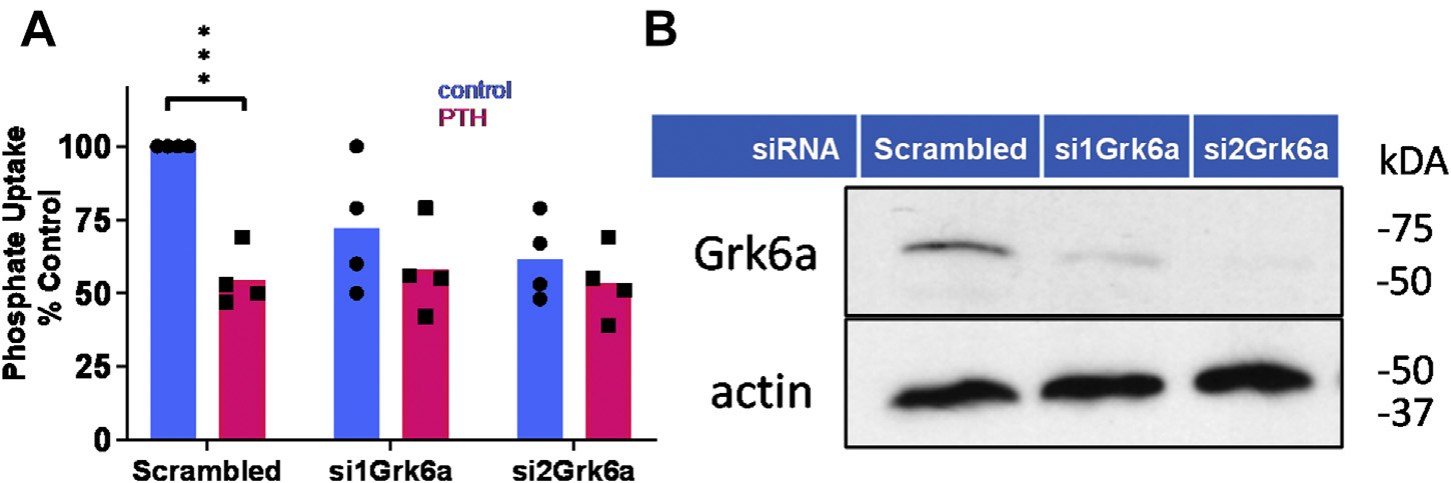
**Grk6a knockdown eliminates PTH-sensitive phosphate uptake.***A*, Grk6a expression was inhibited by siRNAs targeted to opossum Grk6a (si1Grka, si2Grk6a). Phosphate uptake was measured in opossum kidney (OK) cells as described in Experimental procedures. Results report the mean ± SD (*n* = 4; ^∗^^∗^^∗^*p* < 0.001, ANOVA); *B*, confirmation of Grk6a knockdown was assessed by immunoblot and compared with β-actin from the same samples. si1GRK6A and si2GRK6A reduced GRK6A expression by 90% and 98%, respectively, as compared with cells transfected with Scrambled siRNA (*n* = 2). GRK6A, G protein–coupled receptor kinase 6A; PTH, parathyroid hormone.

We first tested whether GRK6A is required for PTH-inhibitable phosphate uptake. For these experiments, we used opossum kidney (OK) cells that constitutively express Npt2a, Nherf1, and Grk6a and are considered the definitive model for PTH-sensitive phosphate transport†. Grk6a was knocked down with targeted siRNA constructs. Both siRNA1 and siRNA2 reduced the response to PTH in the treated cells (Fig. 1*A*). si1Grk6a and si2Grk6a both reduced Grk6a expression by 90% and 98%, respectively (Fig. 1*B*). This finding implicates Grk6a in PTH-mediated signaling and regulation of Npt2a function.

**Figure 2:**
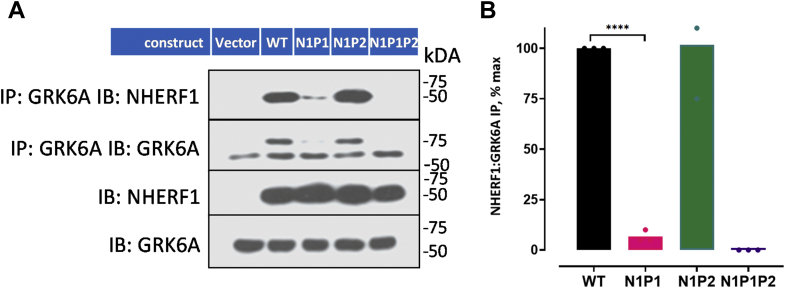
**GRK6A binds PDZ1–NHERF1 under resting conditions**. *A*, representative immunoblot of NHERF1:GRK6A coIP. HA-GRK6A was cotransfected with either empty vector or the indicated FLAG–NHERF1 construct into OKH cells. WT-NHERF1 coimmunoprecipitates with GRK6A, as does N1P2–GAGA–NHERF1 (PDZ1 intact). N1P1–GAGA (PDZ2 intact) and N1P1P2–GAGA/GAGA (both PDZ domains are modified) do not interact with NHERF1. *B*, NHERF1 coIP with GRK6A was quantified in transfected OKH cells and normalized to WT-NHERF1 (100%). N1P2 interacts with GRK6A to a similar extent to WT-NHERF1. Disruption of PDZ1 (N1P1–GAGA, N1P1P2–GAGA/GAGA) eliminated binding of GRK6A. Results report the mean ± SD (*n* = 3; ^∗^^∗^^∗^^∗^*p* < 0.0001, ANOVA). coIP, coimmunoprecipitation; GRK6A, G protein–coupled receptor kinase 6A; NHERF1, Na^+^/H^+^ exchange regulatory factor-1.

We next examined whether NHERF1 coimmunoprecipitates with Grk6a. Opossum kidney clone H (OKH) cells, functionally deficient in Nherf1 (24), were transfected with Flag-NHERF1 (WT) or Flag-NHERF1 constructs where the PDZ core-binding site -GYGF- was mutated to (-GAGA-) in PDZ1 (N1P1-GAGA), PDZ2 (N1P2-GAGA), or both (N1P1P2-GAGA). GRK6A more strongly interacts with N1P2-GAGA than with N1P1-GAGA (Fig. 2, *A* and *B*) (15). The nature of the high molecular weight band (>75 kDa) (Fig. 2*A*), which is not present in host cells, is not known. The binding affinity between recombinant NHERF1, N1P1-GAGA, or N1P2-GAGA constructs was determined by FA using a FITC-labeled 22-residue C-terminal GRK6A peptide (-^553^QRLFSRQDCCGNCSEEELPTRL^576^ [GRK6Act-22]) (Table 1). The findings reinforce the coimmunoprecipitation results (Fig. 2*A*). Full-length NHERF1 and NHERF1 with the modified PDZ2 (N1P2-GAGA) interact with a FITC-labeled human GRK6A C-terminal 22 amino acid peptide, GRK6Act-22, with dissociation constants (*K*_D_) of 5.3 ± 0.7 μM and 3.6 ± 0.2 μM, respectively. The interaction between full-length NHERF1 with the modified PDZ1 (N1P1-GAGA) and GRK6Act-22, is much less evident (Fig. S1, *blue curve*). To rule out the possibility of an effect of NHERF1 self-association on the *K*_D_, we additionally measured *K*_D_ for an N1P1-GAGA construct wherein the C terminus ^355^-FSNL^358^ was mutated to -^355^AAAA^358^ (Δ4). Only a modest effect was observed on the binding between N1P1-GAGAΔ4 and GRK6Act-22 (Fig. S1, *green curve*). However, deleting the NHERF1 C-terminal (ΔEBD, 326–358 aa) substantially increased binding affinity (Fig. S1, *red curve*). The observed effect on affinity corroborates earlier studies showing that the flexible C-terminal EBD of NHERF1 limits access of cystic fibrosis transmembrane conductance regulator (CFTR) (-TRL ligand) to PDZ domains (18).

**Table 1:** Binding affinities between full-length NHERF1 or NHERF1 constructs and GRK6Act-22

NHERF1 construct	*K*_D_, μM^a^
WT NHERF1	5.3 ± 0.7
N1P2-GAGA	3.6 ± 0.2
N1P1-GAGA	>100

FA, fluorescence anisotropy; GRK6Act-22, -^553^QRLFSRQDCCGNCSEEELPTRL^576^; NHERF1, Na^+^/H^+^ exchange regulatory factor-1.

a Dissociation constants (*K*_D_s) were derived from the three independent FA experiments performed in triplicate. The means and SD are given (*n* = 3; ∗∗∗∗*p* < 0.0001, ANOVA).

**Figure 3:**
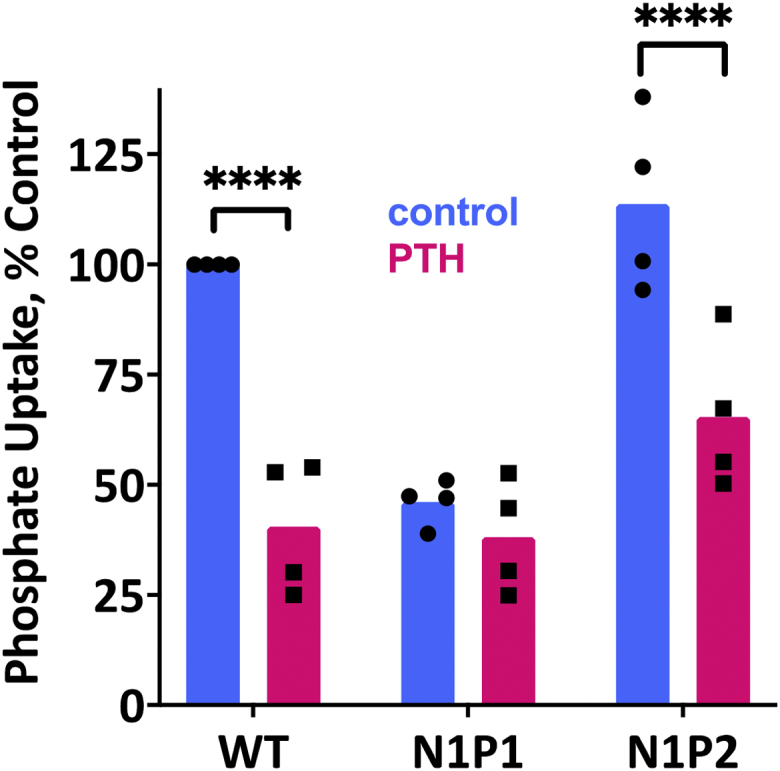
**Effect of NHERF1 PDZ mutants on basal and PTH-sensitive phosphate uptake.** OKH cells were transiently transfected with WT-NHERF1, N1P1-GAGA, or N1P2-GAGA. Cells were treated with vehicle or with 100 nM PTH(1–34). N1P2–GAGA–NHERF1 and WT-NHERF1 support PTH-inhibitable phosphate uptake, whereas N1P1–GAGA does not. Results report the mean ± SD (*n* = 4; ^∗^^∗^^∗^^∗^*p* < 0.0001, ANOVA). NHERF1, Na^+^/H^+^ exchange regulatory factor-1; PTH, parathyroid hormone.

Next, we probed if N1P2-GAGA interacts with GRK6A to support PTH action on phosphate uptake. To accomplish this, we used OKH cells transfected with N1P2-GAGA and treated with 100 nM PTH(1–34). WT NHERF1 and N1P1-GAGA were used as positive and negative controls, respectively. Surprisingly, N1P2-GAGA, like WT NHERF1, supports PTH inhibition of phosphate transport (Fig. 3). These findings imply that despite the modified PDZ2 core-binding motif in N1P2-GAGA, Grk6a nonetheless is able to interact with PDZ2, leading to phosphorylation of Ser^290^ and PTH action. How, then, might Grk6a bind PDZ2 with a disrupted core-binding motif?

Insofar as N1P2-GAGA with the altered PDZ core-binding motif (-^163^GYGF^166^-) is denied a canonical interaction with the Grk6a C-terminal PDZ-ligand motif (-TRL), we sought to determine which residues in N1P2-GAGA support binding with Grk6a. We reasoned that the appearance of phospho-Ser^162^ flanking the PDZ2 core-binding motif (-^163^GYGF^166^-) and known as a direct substrate of PKCα (17, 18) might augment binding of PDZ2 with the -TRL motif of Grk6a.

### Effect of Ser^162^ on PTH-sensitive phosphate transport

**Figure 4:**
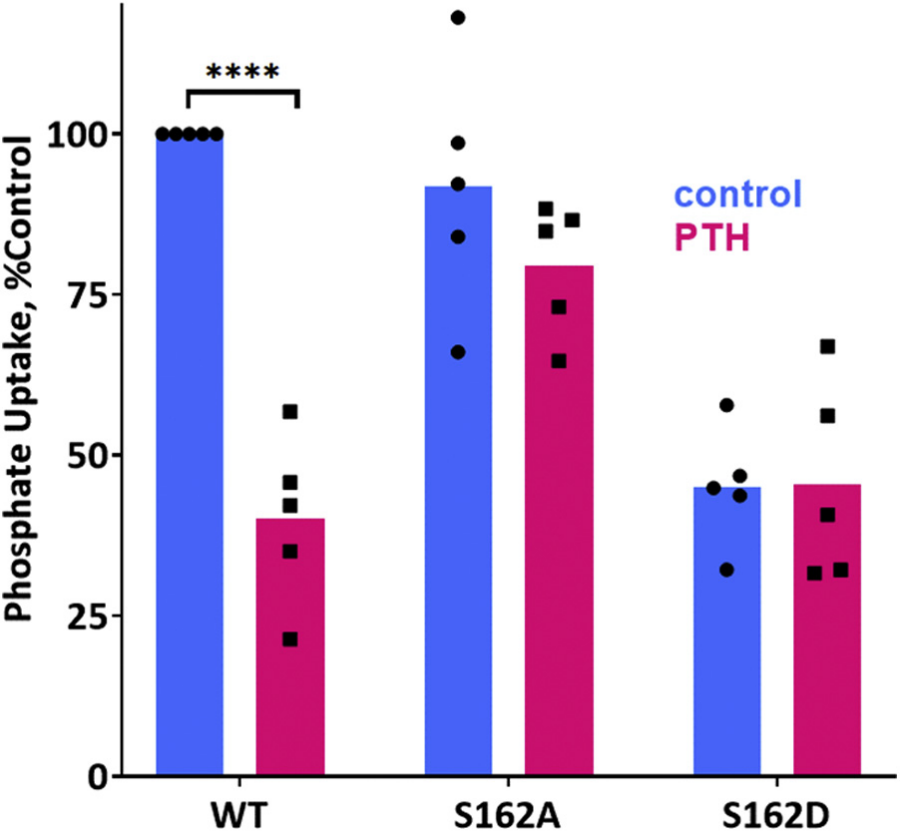
**Ser**^**162**^**is essential for PTH-inhibitable phosphate uptake**. OKH cells were transiently transfected with WT-NHERF1 or with S^162^A-NHERF1 or S^162^D- NHERF1. Cells were treated with vehicle or with 100 nM PTH(1–34). Results report the mean ± SD (*n* = 4; ^∗^^∗^^∗^^∗^*p* < 0.0001, ANOVA). NHERF1, Na^+^/H^+^ exchange regulatory factor-1; PTH, parathyroid hormone.

**Figure 5:**
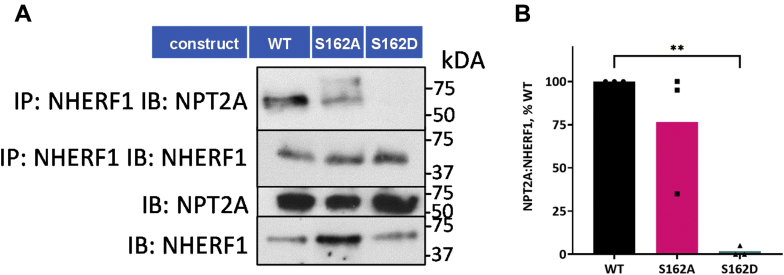
**S**^**162**^**D phosphomimetic mutation disrupts the interaction between NHERF1 and NPT2A**. *A*, about 293 cells were transfected with HA-NPT2A and the indicated form of FLAG–NHERF1 (WT, S^162^A, S162D). Phosphoresistant S^162^A coimmunoprecipitated with NHERF1 nearly as robustly at WT. Notably, the S^162^D phosphomimic ablated the interaction with NPT2A (*n* = 1). Identical results were observed with OKH cells transfected with HA-GFP-NPT2A and FLAG-NHERF1 (WT, S^162^A, S^162^D) (*n* = 2). *B*, the summary of quantified IP results from (*A*). Results were normalized to WT-NHERF1 (100%) and report the mean ± SD (*n* = 3; ^∗^^∗^*p* < 0.01, ANOVA). NHERF1, Na^+^/H^+^ exchange regulatory factor-1.

To investigate the effect of Ser^162^ phosphorylation on PTH-sensitive phosphate transport, we generated NHERF1 constructs wherein Ser^162^ was replaced by Ala (phosphoresistant mutant) or by Asp (phosphomimetic mutant). The phosphomimetic S^162^D mutation resulted in a significant reduction of basal phosphate transport compared with WT NHERF1 (Fig. 4), reflecting the absence of an interaction between Npt2a and NHERF1 S^162^D. In contrast, the phospho-resistant S^162^A mutant did not affect basal phosphate transport but somewhat abrogated the inhibitory response to PTH compared with WT NHERF1 (Fig. 4). Representative immunoblots demonstrate that the S^162^A mutant associates with Npt2a, whereas S^162^D failed to coimmunoprecipitate with Npt2a (Fig. 5). These results suggest that phosphomimetic S^162^D may restrict the interaction between NHERF1 PDZ1 and Npt2a.

### Phospho-Ser^162^ increases association between PDZ2 and GRK6A

To explore further the role of Ser^162^ on GRK6A binding phosphorylated Ser^162^ (hereafter pSer^162^), was genetically introduced in a recombinant PDZ2 (133–300) using amber codon suppression (25). Previously, semisynthesis was effectively applied to generate site-specific phosphorylated PDZ domains (26). Here, we used amber codon suppress ion to genetically encode pSer at position 162, which in the future will allow us to access phosphorylated full-length proteins comprising PDZ domains. This strategy requires a unique aminoacyl synthetase and tRNA pair specific for pSer and orthogonal to the natural translation system of *Escherichia coli*, thereby preventing cross-reactivity (25). Upon induction with IPTG, pSer was site-specifically incorporated into PDZ2 at position 162. The introduction of pSer was characterized as a mass change of 80 Da corresponding to the phosphate group as determined by LC-MS (Fig. S2). The secondary structure of the protein was measured by CD (Fig. S2).

The binding affinity between pSer^162^-PDZ2 (133–300 aa) and FITC-labeled GRK6Act-22 was determined by FA and compared with the WT PDZ2 domain (133–300 aa) and S^162^A-PDZ2 (133–300 aa) (Table 2). The results demonstrate that the *K*_D_ values for the interaction between WT PDZ2 or S^162^A-PDZ2 and GRK6Act-22 were comparable (51.3 ± 0.4, 43.4 ± 0.4 μM) and 2-fold higher than for pSer^162^-PDZ2 (26.1 ± 0.8; *p* < 0.0001 ANOVA) (Table 2 and Fig. S3). Thus, pSer^162^-PDZ2 enhanced binding affinity for GRK6A.

**Table 2:** Binding affinities between isolated PDZ2 domain or PDZ2 mutants and GRK6Act-22

PDZ2 construct	*K*_D_, μM
WT PDZ2	51.3 ± 0.7^a^
pSer^162^-PDZ2	26.1 ± 0.8^b^
S^162^A-PDZ2	43.4 ± 0.4^a^

FA, fluorescence anisotropy; GRK6Act-22, -^553^QRLFSRQDCCGNCSEEELPTRL^576^; pSer^162^, phosphorylated Ser^162^.

Phosphorylated design is indicated by “p”. FA experiments were performed in triplicate.

a Means and SDs are given (*n* = 3; ∗∗∗∗*p* < 0.0001, ANOVA).

b Means and SDs are given (*n* = 5; ∗∗∗∗*p* < 0.0001, ANOVA).

### Computational prediction of NHERF1 and GRK6A binding determinants

We then applied explicit-solvent MD simulation to explore the structural determinants underlying the binding specificity of NHERF1 PDZ domains for GRK6A. A 9-residue, C-terminal GRK6A peptide (-^568^SEEELPTRL^576^) was used. The complexes between PDZ1, PDZ2, or pSer^162^-PDZ2 and a 9-residue, C-terminal GRK6A peptide (-^568^SEEELPTRL^576^) were computationally generated with the docking program ZDOCK (27) as described in Experimental procedures. The docking structures (Fig. S4) demonstrate that the C terminus of GRK6A (-T^−2^R^−1^ L^0^) goes deep in the PDZ1 or PDZ2 hydrophobic cavity between the α2 helix and the β2 sheet. The -TRL motif is involved in canonical interactions with the conserved residues from the core-binding motif ^23^GYGF^26^, α2 (Val^76^, Arg^80^), β2 (Leu^28^), or ^163^GYGF^166^, Val^216^, Arg^220^, Leu^168^ of PDZ1 and PDZ2, respectively. His^72^ or His^212^ of PDZ1 and PDZ2, respectively, specifically target Thr^−2^ of GRK6A. The PDZ2–GRK6Act-9 peptide (-^568^SEEELPTRL^576^) (hereafter GRK6Act-9) complex, in contrast to PDZ1–GRK6A, was unstable and showed a tendency to dissociate after 130 ns of MD simulation. MD simulations predict that in addition to the canonical PDZ-ligand interactions, Glu^43^ of PDZ1 associates tightly with the positively charged side chain of Arg^−1^ of GRK6A. The short distance (less than 2 Å) between the carboxylate oxygens of Glu^43^ (Oε^1^ and Oε^2^) and the NHη^2^ group of Arg^−1^ of GRK6A permits formation of strong electrostatic interactions (Fig. S4*A*). Compared with Glu^43^, Asp^183^ at the homologous position in PDZ2 does not interact with the side chain of Arg^−1^. The latter is solvent-exposed and characterized by multiple orientations during the MD simulation. The distances between the NHη^2^ group of Arg^−1^ and carboxylate oxygens of Asp^183^ (Oδ^1^ and Oδ^2^) from 6.8 to 13.4 Å along the simulation time trajectory (Fig. S4*B*) indicate that the naturally occurring Glu to Asp replacement on PDZ2 decreases the binding affinity for the C-terminal motif of GRK6A. This observation is consistent with coimmunoprecipitation (Fig. 2) and FA experimental data (Table 1 and Fig. S1) showing a weak association between PDZ2 and GRK6A compared with PDZ1. A similar tendency was observed for NHERF1 PDZ domains interacting with the C-terminal -TRL motif of NPT2A (28) or the C-terminal -TRL motif of CFTR (29, 30, 31).

**Figure 6:**
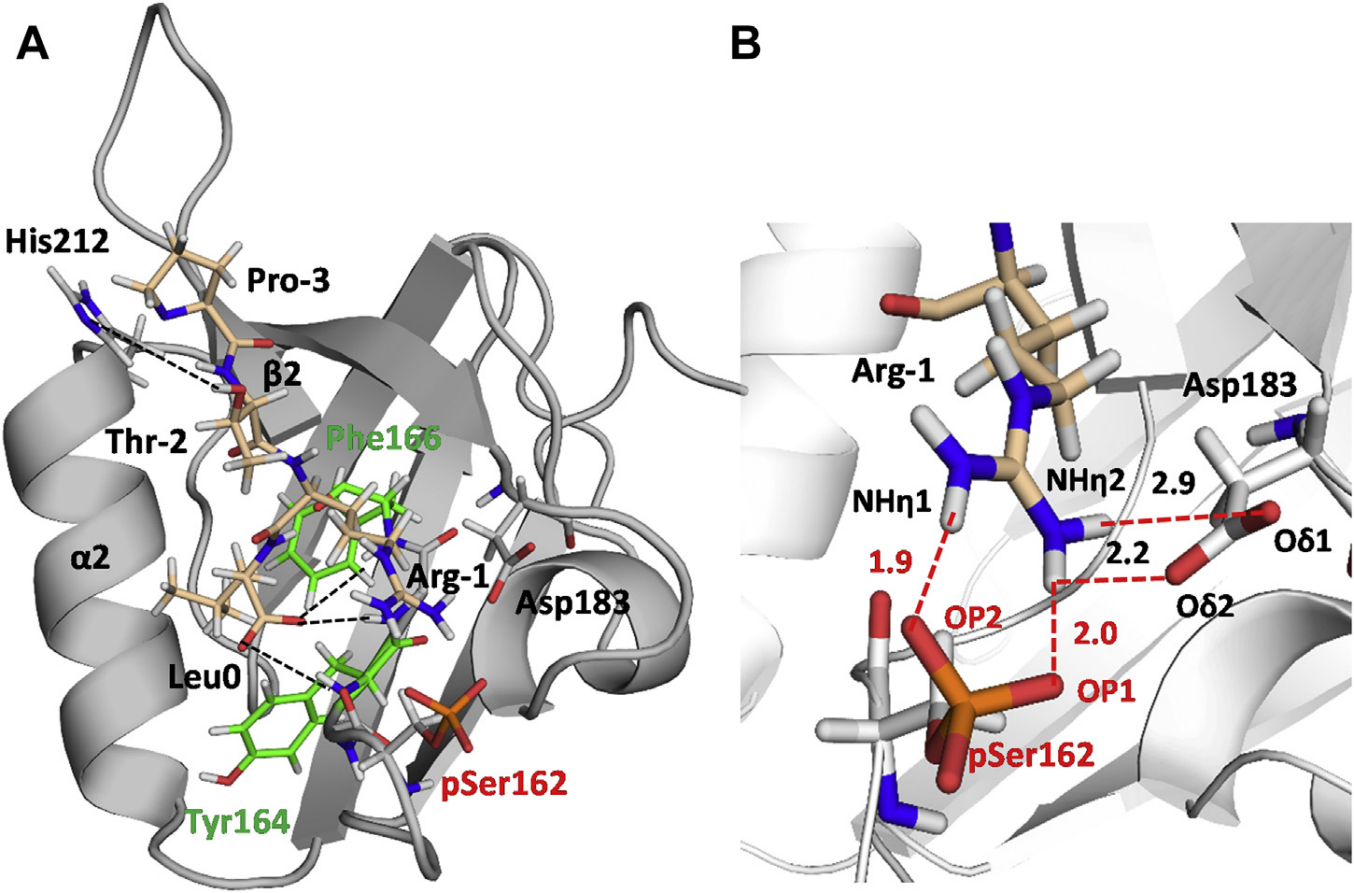
**Computational model of pSer**^**162**^**-PDZ2 bound to the GRK6Act-9 peptide.** The PDZ2 domain is highlighted in *gray cartoon*, whereas the GRK6Act-9 peptide is represented in *wheat sticks*. *A*, the extreme C terminus of GRK6Act-9 (-P^−3^T^−2^R^−1^ L^0^) is inserted in the binding pocket of pSer^162^-PDZ2 between the α2 helix and the β2 sheet. Tyr^164^ and Phe^166^ forming the hydrophobic pocket are shown as *green sticks*. Hydrogen bonds between the backbone amide (NH) of Tyr^164^, Gly^165^, and Phe^166^ and C-terminal oxygens of Leu^0^ and between His^212^ and Thr^−2^ are shown as *black dotted lines*. The five residues from the N-terminal end of the GRK6Act-9 peptide are facing outside of the PDZ-binding pocket and omitted for simplicity. *B*, the plausible electrostatic network involved pSer^162^-Arg^−1^-Asp^183^ is shown as *red dotted lines*. Average distances between OP1 of pSer^162^ and NHη^1^ of Arg^−1^ or between OP2 and NHη^2^ are 2.0 Å and 1.9 Å, respectively and between NHη^1^ of Arg^−1^ and Oδ^1^ or Oδ^2^ of Asp^183^ are 2.9 Å and 2.2 Å, respectively. Distances were calculated along the last 10 ns of MD simulation. Hydrogen atoms are *white*, oxygen atoms are *red*, and nitrogen atoms are *blue*. GRK6Act-9, -^568^SEEELPTRL^576^.

MD simulations demonstrate that incorporation of phospho-Ser^162^ does not induce conformational changes in the PDZ2–GRK6Act-9 structure. However, comparative analysis of PDZ2–GRK6Act-9 and pSer^162^–PDZ2–GRK6Act-9 revealed a significant difference in the orientation of Arg^−1^ of the GRK6A -TRL motif (Fig. 6 and Fig. S4). The positively charged side chain of Arg^−1^ rotates toward the negatively charged phosphate group of Ser^162^ during the first 2 ns of MD simulation and remains in this conformation for the remainder of the simulation (159 ns) (Fig. 6). The average distances calculated between the NHη^1^ or NHη^2^ group of Arg^−1^ of GRK6A and OP1 or OP2 group of pSer^162^ are around 2 Å for the last 10 ns of MD simulation (Fig. 6). This indicates that the negatively charged phosphate group has a significant impact on dynamics and conformation of the positively charged side chain of Arg^−1^. Remarkably, the side chain of Asp^183^ changes its orientation and moves toward the side chain of Arg^−1^. The average distances calculated between the NHη^2^ group of Arg^−1^ of GRK6A and the carboxylate oxygens of Asp^183^ (Oδ^1^ and Oδ^2^) of pSer^162^-PDZ2 for the last 10 ns of MD simulation (2.2 Å and 2.9 Å, respectively) (Fig. S5) suggest a strong stabilizing effect of phospho-Ser^162^ on the electrostatic interaction of Arg^−1^ with Asp^183^. We further computationally substituted Tyr^164^ and Phe^166^ to Ala in the carboxylate-binding site of pSer^162^-PDZ2. MD simulations (100 ns) demonstrate that the N-terminal part of the GRK6Act-9 peptide is solvent-exposed and faces out of the binding cavity. However, the extreme C terminus and the side chain of Arg^−1^ of GRK6Act-9 remains in the same conformation as observed for pSer^162^-PDZ2 (Fig. S6). Substitution of Tyr^164^ and Phe^166^ by Ala disrupts the hydrophobic network formed by side chains of Tyr^164^, Phe^166^, and Leu^0^ but does not disrupt interactions between backbone amides (NH) of Tyr^164^Ala, Gly^165^, and Phe^166^Ala and C-terminal oxygens of Leu^0^ or a hydrogen bond between His^212^ and Thr^−2^ (Figs. 6 and S6). MD simulations predict that the positively charged side chain of Arg^−1^ is in a dynamic equilibrium between the two negatively charged side chains, pSer^162^ and Asp^183^. Thus, pSer^162^ has a significant electrostatic impact on the stabilization of the side chain of Arg^−1^ and the formation of the pSer^162^-Arg^−1^-Asp^183^ network (Figs. 6 and S6).

## Discussion

**Figure 7:**
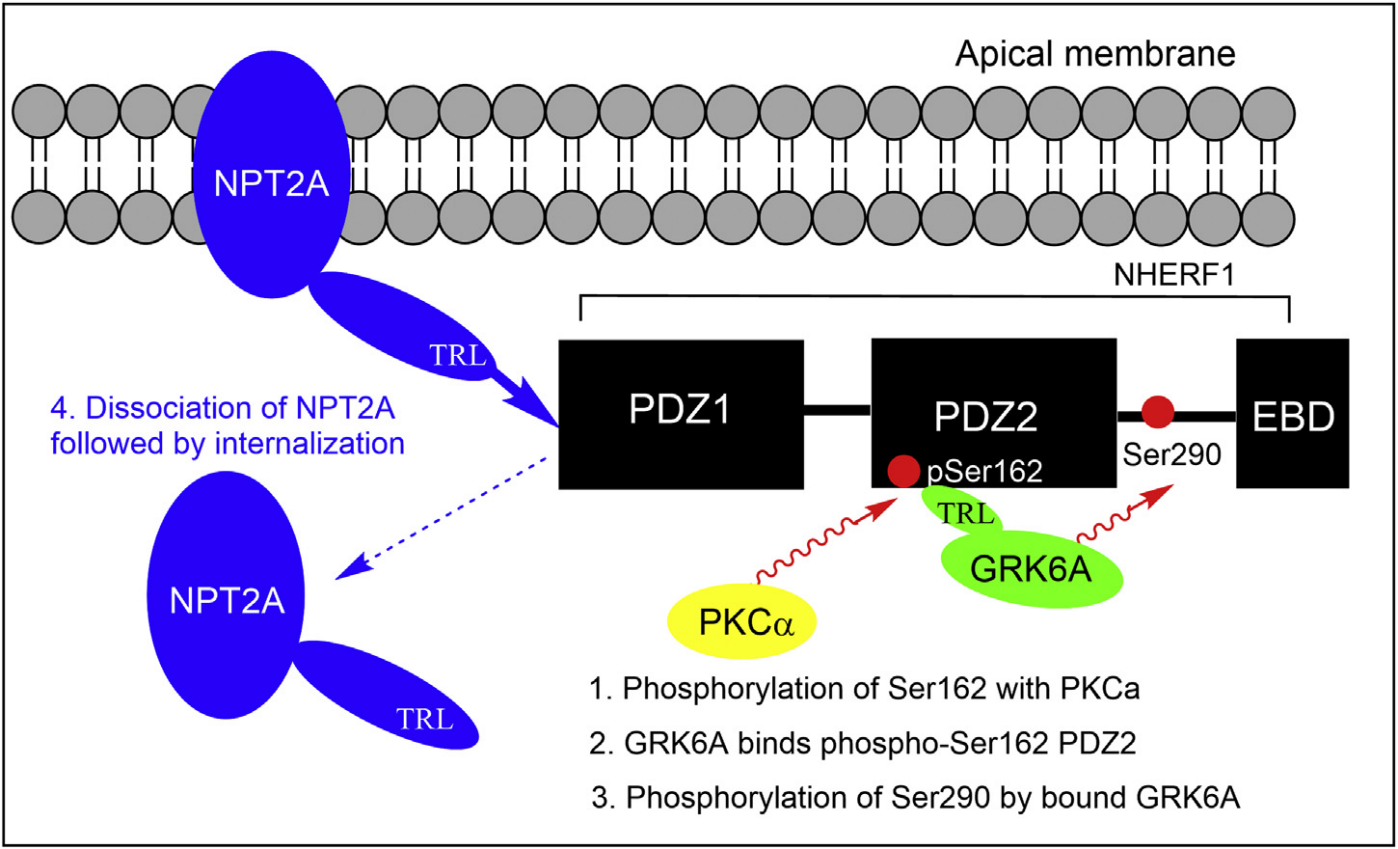
**General scheme represents the order of events along NHERF1-dependent PTH-sensitive phosphate uptake.** NHERF1 PDZ1 binds NPT2A through the C-terminal (-TRL) motif and that keeps NPT2A at the apical membrane. PTH-induced phosphorylation promotes phosphorylation of Ser^162^ by PKCα. GRK6A binds pSer^162−^PDZ2 through the C-terminal (-TRL) motif and phosphorylates Ser^290^. NPT2A dissociates from NHERF1 and internalizes. GRK6A, G protein–coupled receptor kinase 6A; NHERF1, Na^+^/H^+^ exchange regulatory factor-1; PTH, parathyroid hormone.

Although NHERF1 PDZ domains are very similar, they have a distinct ligand specificity. NHERF1 PDZ1 interacts with the type-2 sodium-phosphate cotransporter NPT2A (*SLC34A1*) (32, 33) via C-terminal PDZ-ligand motif (-TRL^639^). This association is required for hormone-regulated phosphate transport and proper localization of NPT2A at the apical membrane (Fig. 7). Previously, we and others showed that the specificity of the interaction between NHERF1 and target ligands harboring Arg^−1^ at C terminus depend on Glu^43^ of PDZ1 (12, 28, 31, 34). Glu^43^ coordinates the interaction with Arg^−1^ of the C-terminal recognition motif of NPT2A (-T^−2^R^−1^L^0^) and establishes the strong electrostatic network required for hormone-sensitive phosphate transport (4, 28). Asp^183^ located at the homologous position in PDZ2 is shorter than Glu^43^. The current MD simulations, prior study (28), and available NMR structure (31) demonstrate that the side chain of Asp^183^ is flexible and not involved in a stable interaction with Arg^−1^. The Glu^43^Asp mutation in PDZ1 leads to dramatic loss in affinity with the similar C-terminal -TRL motif of CFTR (31). Consistently, an Asp^183^Glu rescue mutation in PDZ2 restores the electrostatic network between Asp^183^Glu and Arg^−1^ specific for PDZ1 and significantly increases the binding affinity of PDZ2 (28).

In the present study, we show that knocking down Grk6a by si1RNA or si2RNA blocks Npt2a-dependent phosphate uptake in response to PTH (Fig. 1). This observation indicates that GRK6A is an essential regulatory component of NPT2A-dependent PTH-sensitive phosphate transport and corroborates previous findings that GRK6A pharmacological inhibitors abolish PTH action (16). Our data agree with an earlier study demonstrating that NHERF1 is constitutively phosphorylated at Ser^290^ (35). As expected, siRNA-GRK6A–treated cells do not support basal phosphate uptake (Fig. 1). GRK6A, similar to NPT2A, associates with NHERF1 PDZ domains via the C-terminal PDZ-ligand motif (-TRL). Compatible binding affinities (3 μM to 5 μM) were observed for NHERF1–GRK6Act-22 (Table 1) and NHERF1–NPT2Act-22 (28) association. NHERF1 is highly concentrated at apical membranes (8). The concentration of NHERF1 [P] is in great excess over total membrane-delimited NPT2A [Nt] or GRK6A [Gt]. At the condition when [P] >> [Nt] and [P] >> [Gt], equations for the equilibrium binding are as follows:

(1) $$\left[\text{PG}\right]=\;\left[\text{P}\right]\times \left[\text{Gt}\right]/{\text{K}}_{\text{D}2}\;+\;\left[\text{P}\right]$$

(2) $$\left[\text{PN}\right]=\;\left[\text{P}\right]\times \left[\text{Nt}\right]/{\text{K}}_{\text{D}1}\;+\;\left[\text{P}\right]$$

Where [PG], [P], [Gt], [PN], and [Nt] are the concentrations of PDZ1–GRK6A, PDZ1, GRK6A (total), PDZ1–NPT2A, and NPT2A (total), respectively. *K*_D1_ and *K*_D2_ are dissociation constants for the PDZ1–GRK6A and PDZ1–NPT2A complexes, respectively. These equations were solved by dividing both sides of Equation 1 by Equation 2. Assuming that *K*_D1_ ∼ *K*_D2_ yields [PG]/[PN] = [Gt]/[Nt]. When [Nt] >> [Gt], then [PG]/[PN] → 0. When [P] → ∞, PDZ1 occupancy will be determined by the total concentration of NPT2A [Nt] although NPT2Act-22 (28) and GRK6Act-22 have similar binding affinities for PDZ1 (Table S1).

The observed weak interaction between isolated PDZ2 and GRK6Act-22 (*K*_D_ = 51.3 μM in Table 2), as previously reported (15), is consistent with data obtained for PDZ2 in full-length NHERF1 (N1P1–GAGA in Fig. 2*A* and Table 1). However, this interaction is physiologically relevant and obligatory for the constitutive cellular (15), PTH-induced phosphorylation of Ser^290^ in NHERF1 (Fig. 7), and NHERF1-dependent PTH-sensitive phosphate transport (16). Generally, the interaction between the carboxylate-binding site of the PDZ domain and the C-terminal residues of the target ligand control binding. To characterize functional and molecular details of the interaction between PDZ2 and GRK6A, we mutated Tyr^164^ and Phe^166^ to Ala in the carboxylate-binding loop of NHERF1 (GYGF→ GAGA). Published studies established that substitution of bulk hydrophobic residues (Tyr, Phe, Ile, or Leu) in the carboxylate binding loop of PDZ domains by Ala disrupts the hydrophobic network, making PDZ-ligand interactions very weak and frequently unrecognized (4, 20, 36, 37). Mutation of core-binding Tyr^164^ and Phe^166^ in NHERF1 PDZ2 (N1P2-GAGA) decreased but did not block phosphate transport in response to PTH (Fig. 3). This finding strongly suggests that the PDZ2 domain retains its ability to interact with the C terminus of GRK6A *in vivo.* This raised the hypothesis that unrecognized binding determinants might stabilize the association between PDZ2 and GRK6A. Analysis of residues that may contribute to the binding pointed to Ser^162^, known as a PKCα phosphorylation site in human NHERF1 (Fig. 7) (17, 18). Notably, NHERF1 homologs (mouse, rabbit) harbor asparagine at the corresponding position. PKCα action is unique for PDZ2 inasmuch as PDZ1 has Asn^22^ at the homologous position. The latter may affect PDZ1 binding affinity (12). Ser^162^ at the corresponding position of PDZ2 also may stabilize Arg^−1^ through backbone interactions (38). In this instance, replacement of Ser^162^ by Ala would not be expected to alter the binding affinity between PDZ2 and the C terminus of GRK6A as reported here (Table 2). The current results suggest that introducing the double negatively charged phosphate group at Ser^162^ increases the binding affinity between PDZ2 and GRK6A (Table 2). Nonetheless, it is not intuitive that the effect of pSer^162^ on binding is sufficient albeit relatively minor. One possible explanation is that pSer^162^-PDZ2 was generated and purified using a different protocol compared with recombinant WT or S^162^A-PDZ2. Experimental conditions (buffer, concentration, incubation time, titration) can affect FA measurements. The binding affinity was determined for the isolated PDZ2 domain bound a relatively short (22 aa) C-terminal peptide of GRK6A. We speculate that binding determinants beyond the C-terminal motif of GRK6A may also contribute and stabilize the interaction with full-length NHERF1 *in vivo*. Overall, biochemical experiments support the view that Ser^162^ is required for regulation of NPT2A-mediated hormone-sensitive phosphate transport, and specificity of the PDZ2 domain for GRK6A is not determined by the conserved subset of residues (Tyr^164^ and Phe^166^) but rather specific pSer^162^. This finding corroborated with our prior studies demonstrated that a small change in the free energy of binding characterizes PDZ-ligand specificity and is attributed to the enthalpy–entropy compensation (28, 39).

We further reasoned that phosphoresistant NHERF1 S^162^A should reduce PTH action and phosphate transport (Fig. 4). The partial inhibition of PTH-sensitive phosphate transport (Fig. 4) relates to constitutive phosphorylation of Ser^290^ (15, 16). This observation is compatible with prior work showing that the low binding between the PDZ2 K^158^A-K^159^A mutant and Grk6a concurrently lowers Ser^290^ phosphorylation (15). In accord with this finding, S^162^A diminishes the amount of GRK6A bound to NHERF1 and decreases phosphate uptake (Fig. 4). As anticipated, the phosphoresistant S^162^A substitution does not impede the interaction between NHERF1 and NPT2A (Fig. 5). Surprisingly, phosphomimetic NHERF1 S^162^D did not coimmunoprecipitate with NPT2A (Fig. 5) and suppressed PTH action (Fig. 4). Intriguingly, a similar tendency was observed for Nherf1 Ser^77^ and Thr^95^ phosphorylation sites located in PDZ1 (40). S^77^D blocks interaction with Npt2a and basal phosphate uptake, whereas T^95^A inhibits PTH-sensitive phosphate transport. We speculate that replacement of OH-Ser^162^ by COOH-S^162^D causes a conformational reorganization of NHERF1 and, in particular, in the linker segments connecting PDZ1 and PDZ2 (112–146 aa) and PDZ2 and the EBD (252–358 aa), resulting in an NHERF1 conformation that inhibits association between PDZ1 and NPT2A, thereby terminating PTH action.

The biochemical data provided here indicate that the binding affinity of PDZ2 is regulated by phosphorylation of Ser^162^ (Table 2). MD simulations complement and provide additional details about the interaction between pSer^162^-PDZ2 and the -T^−2^R^−1^L^0^ motif of GRK6A. The side chain of Arg^−1^ emerged rapidly with the negatively charged phosphate group of Ser^162^. The side chain of Asp^183^, facing the bulk water in PDZ2, was rotated toward Arg^−1^ in pSer^162^-PDZ2. The MD simulation demonstrates that introducing the phosphate group faithfully mimics enzymatic phosphorylation and promotes local conformational changes. The formation of an electrostatic network of pSer^162^-Arg^−1^-Asp^183^ is vital for stabilizing the pSer^162^–PDZ2–GRK6A complex (Table 2 and Fig. S3). This observation is entirely compatible and consistent with our earlier studies that underlined a limited contribution of Asp^183^ on the binding with the C-terminal -TRL motif of NPT2A (28). The C-terminal -TRL motif is not unique. CFTR with the same C-terminal -TRL motif (29, 41) as GRK6A and NPT2A is another natural partner of NHERF1 (42). A recently published X-ray structure complemented by MD simulations (50 ns) demonstrate that Arg^−1^ of CFTR can form a salt bridge with Asp^183^-PDZ2 (38). However, much longer MD simulation is required to estimate dynamics of this interaction. Further crystallization of NHERF1 PDZ domains with the C-terminal -TRL motif of NPT2A is necessary to distinguish binding determinants and improve our understanding of NHERF1 PDZ-ligand specificity.

Site-specific incorporation of pSer^162^ applied here for NHERF1 PDZ2 introduces an analogous but not identical functional group compared with phosphomimetic mutagenesis. The phosphate group has a −2 negative charge compared with the single negative charge of the Asp carboxylate group. The bulkier phosphate group compared with the carboxylate group may importantly perturb the local protein structure (43). This observation underscores the limitation and potential hazard of using phosphomimetics to draw conclusions about phosphorylation and demonstrates the strength of site-specific introduction of pSer using amber codon suppression. Together, these ensemble factors are critical for identifying protein–ligand interactions *in vitro*. Rearrangement of an electrostatic network in pSer^162^-PDZ2 and changes in side chain dynamics may allosterically regulate interdomain communication in NHERF1, leading to phosphorylation of Ser^290^ in its EBD and concurrent disassembly of NPT2A from PDZ1 (Fig. 7), which is required for inhibition of phosphate transport (16). Allosteric networks and allosteric communication are essential regulators of PDZ-containing multidomain protein conformational changes and function (44, 45).

In summary, the present findings provide strong evidence that GRK6A is vital for hormone-sensitive phosphate uptake. Our biochemical results demonstrate that phosphorylation of Ser^162^ regulates the interaction between PDZ2 and the GRK6A C-terminal PDZ-binding motif. This observation is corroborated by binding affinities and MD simulations. Based on these outcomes, we propose an extended model of PTH-sensitive phosphate transport where NHERF1 PDZ1 associates with NPT2A and serves as a functional platform for hormone-regulated phosphate transport, whereas PDZ2 targets GRK6A via PKCα phosphorylation of Ser^162^ and works as a regulatory domain. GRK6A and PKCα represent two complementary and parallel pathways of PTH signaling. PTH-induced phosphorylation of Ser^162^ may be part of a selectivity mechanism that allows NHERF1 PDZ domains to discriminate between target ligands.

## Experimental procedures

### Cell culture and transfection

Parental OK or NHERF1-deficient (OKH) cells (4) were grown in Dulbecco's modified Eagle's medium/Ham's F-12 50:50 medium (Mediatech, 10-090-CV) supplemented with 10% fetal bovine serum and 1% pen/strep.

Cells were transfected with the indicated plasmids using jetPRIME (Polyplus) or Lipofectamine 3000 (Invitrogen) according to the manufacturer's instructions. Stable OKH cells expressing FLAG–NHERF1, FLAG–NHERF1 constructs or FLAG–NHERF1 mutants, and HA-GRK6A were prepared by transferring cells and then selecting for stable expression with puromycin or G418 and then subcloning by limiting dilution.

### Preparation of endogenous NHERF1 and NHERF1 constructs

WT or mutant NHERF1 tagged with FLAG was prepared as described (16).

### Phosphate uptake

OKH cells were seeded on 12-well plates and 24 h later were transfected with FLAG–NHERF1 (WT or mutant) using jetPRIME, as indicated. After 48 h, the cells were serum-starved overnight. The next day, the transfected cells were treated with 100 nM PTH(1–34) or water (vehicle) in cell culture media for 2 h. The hormone-supplemented medium was aspirated, and the wells were washed 3 times with 1 ml of Na-replete buffer (140 mM NaCl, 4.8 mM KCl, 1.2 mM MgSO_4_, 0.1 mM KH_2_PO_4_, 10 mM Hepes, pH 7.4). The cells were incubated with 1 μCi of ^32^P orthophosphate (PerkinElmer, NEX053) in 1 ml of the Na-replete wash buffer for 10 min. Phosphate uptake was terminated by placing the plate on ice and rinsing the cells three times in Na-free wash buffer (140 mM N-methyl-D-glucamine, 4.8 mM KCl, 1.2 mM MgSO_4_, 0.1 mM KH_2_PO_4_, 10 mM Hepes, pH 7.4). The cells in each well were extracted using 500 μl 1% Triton X100 (Sigma Cat X100) in water overnight at 4 °C. A 250 μl aliquot was counted in a Beckmann Coulter LS6500 liquid scintillation counter. Data were normalized, where 100% was defined as cpm of phosphate uptake under control conditions.

### siRNA knockdown

siRNA for Grk6a knockdown was designed by and purchased from Integrated DNA Technologies (IDT). The siRNA was transfected into OK cells using LipoJet (100468, SignaGen Laboratories). Cells were transfected on 60 mm dishes. Forty-eight hours after transfection, the cells were trypsinized and passaged onto 12-well plates for phosphate-uptake assays. Protein lysates were extracted from a 100 μl aliquot of these cells to assess by immunoblot the extent of Grk6a knockdown.

### Immunoprecipitation and immunoblotting

Transfected OKH cells were lysed with 1% Nonidet P-40 (50 mM Tris, 150 mM NaCl, 5 mM EDTA, 1% Nonidet P-40) supplemented with protease inhibitor mixture I (Calbiochem). Lysis was performed for 15 min on ice. Solubilized materials were resolved on 10% SDS–polyacrylamide gels and transferred to Immobilon-P membranes (Millipore) using the semidry method (Bio-Rad). Membranes were blocked overnight at 4 °C with 5% nonfat dried milk in Tris-buffered saline plus Tween 20 (TBST) and incubated with the indicated primary antibodies overnight at 4 °C. The membranes were washed four times for 10 min in TBST and then incubated with goat anti-rabbit IgG or anti-mouse IgG conjugated to horseradish peroxidase at a 1:5000 dilution for 1 h at room temperature. Membranes were washed 4 times for 10 min in TBST. Protein bands were detected by Luminol-based enhanced chemiluminescence (EMD Millipore WBKLS0500).

### Recombinant constructs and protein purification

The expression plasmids pET16-N1P1 encoding PDZ1 (1–140) and pET16-N1P2 encoding PDZ2 (133–300 aa) of NHERF1 were kindly provided by Dr Dale F. Mierke (Department of Chemistry, Dartmouth College, Hanover, NH). Plasmid fidelity was confirmed by DNA sequencing (ABI PRISM 377, Applied Biosystems) and subsequent sequence alignment (NCBI BLAST) with human NHERF1 (GenBank AF015926) to ensure the accuracy of the constructs. Recombinant proteins were expressed in *E. coli* BL21 (DE3) cells (Novagen) and purified using Ni-NTA-agarose (Qiagen) (46). Full-length NHERF1 and NHERF1 constructs were generated in the laboratory. The resulting proteins were divided into aliquots and stored in the phosphate buffer (25 mM NaH_2_PO_4_, 10 mM NaCl, pH 7.4) at −80 °C until used for FA experiments. The -GYGF- core-binding mutations were introduced in PDZ1 or PDZ2 of full-length NHERF1 (N1): N1P1: ^23^GYGF^26^/^23^GAGA^26^; N1P2: ^163^GYGF^166^/^163^GAGA^166^; and N1P1P2: ^23^GYGF^26 163^GYGF^166^/^23^GAGA^26 163^GAGA^166^, using the QuikChange site-directed mutagenesis kit. All construct sequences were confirmed by DNA sequencing.

Substitution of Tyr^24^/Tyr^164^ and Phe^26^/Phe^166^ by Ala in the carboxylate binding site of full-length NHERF1 eliminates canonical hydrophobic interactions and therefore significantly decreases binding affinity of PDZ1–GAGA or PDZ2–GAGA to bind PDZ-binding motifs of targets compared with unmodified PDZ domains. NHERF1 with modified carboxylate-binding sites may serve as a suitable model to estimate the interaction between full-length NHERF1 (1–358 aa) where one PDZ domain is available for binding, whereas the other PDZ domain has a negligible binding affinity for binding the target.

### Generation of pSer^162^–PDZ2

Chemically competent *E. coli* BL21 Δ*SerB* (AddGene #34929) cells were cotransformed with pCDF-1b Nherf1 PDZ2 (133–300) Ser^162^TAG 6xHis and pKW2 EF-Sep (25) and plated on LB agar plates containing 100 μg/ml spectinomycin and 25 μg/ml chloramphenicol. Precultures were grown overnight at 30 °C and diluted to an absorbance of 600 nm of 0.1 in fresh LB media containing antibiotics and grown at 37 °C to an absorbance of 600 nm of 0.5. Protein expression was induced by addition of 0.5 mM IPTG, and the medium was supplemented with 1 mM *O*-phospho-L-serine (Sigma). The protein was expressed at 30 °C for 4 h, shaking at 150 rpm. Cells were harvested by centrifugation, 10,000*g*, at 4 °C for 10 min. The cell pellet was lysed in 4 ml/g pellet B-PER Bacterial Extraction Reagent supplemented with DNase and protease inhibitor and spun down by centrifugation, 30,000*g*, at 4 °C for 30 min. The supernatant was loaded on a pre-equilibrated (50 mM Tris, 150 mM NaCl, 2 mM β-mercaptoethanol, pH 8) HisTrap Ni^2+^ column (GE Healthcare), which was extensively washed after loading. The protein was eluted using an ÄKTAExplorer 100 Air system (GE Healthcare) with a gradient of imidazole (50 mM Tris, 150 mM NaCl, 100 mM imidazole, 2 mM β-mercaptoethanol, pH 8). The protein was further purified by gel filtration (50 mM Tris, 150 mM NaCl, 2 mM β-mercaptoethanol, pH 8). The protein concentration was determined by 280 nm absorbance on a Nanodrop 1000 (Thermo Fisher Scientific). The purified protein was analyzed by ESI-LC-MS (Agilent) and Acquity UPLC (Waters) for the identification of mass and purity, respectively. The various purification steps were followed by SDS-PAGE.

### CD

The secondary structure of NHERF1 PDZ2 pSer^162^ 6× His was visualized by CD in 50 mM NaPi buffer, pH 8. All recordings were made using a Jasco J1500 CD spectrometer (Jasco) in 1 mm quartz cuvettes (BioLab). Recordings were carried out on 10 μM protein samples at 25 °C, and at 95 °C immediately followed by a 25 °C scan to assess the refolding. Three accumulated scans for each temperature were acquired for 260 to 190 nm and analyzed in Prism (GraphPad).

### Peptides

A FITC-labeled human GRK6A C-terminal 22 amino acid peptide, GRK6Act-22, was synthesized by LifeTein. The WT and FITC-labeled peptides were dissolved and serially diluted in PBS (pH 7.4).

### FA saturation binding

Solution phase direct binding assays were performed to measure binding affinity (*K*_D_) between NHERF1, NHERF1 constructs (N1P1-GAGA and N1P2-GAGA), isolated PDZ domain, pSer^162^-PDZ2 or S^162^A-PDZ2, and FITC-GRK6Act-22 after the previously reported protocols for NHERF1 PDZ domains (28, 29, 39). All measurements were performed in PBS (pH 7.4) supplemented with 0.1% BSA and 1-mM DTT by applying increasing amounts of the recombinant proteins to a fixed concentration of the FITC-labeled peptide (0.4–1.0 μM). FA assays were run on a 96-well format and performed in triplicate in three to five independent experiments. Polarized fluorescence intensities were measured at 23 °C with a PerkinElmer Wallac VICTOR3 multilabel counter using excitation and emission wavelengths of 485 nm and 535 nm, respectively. Experimental data were analyzed using Prism (GraphPad). All measurements are reported as FA rather than polarization. FA was computed using Equation 1 from the measured fluorescence emission intensities that are polarized parallel (I_‖_) and perpendicular (I_⊥_) to the plane of the incident light (47):

(3) $$\text{r}={\text{I}}_{\text{x}}-{{\text{I}}_{⊥}/\text{I}}_{x}+{2\text{I}}_{⊥}$$

The equilibrium dissociation constant (*K*_*D*_) for the interaction between the indicated PDZ domain and labeled peptide was determined by fitting the FA data to a quadratic equation (47) as described (28).

### Model setup and molecular docking

The structures of the NHERF1 PDZ1 and PDZ2 domains (residues 13–91 and 148–240, respectively) were derived from our previous MD simulations (39, 48). The residues forming the binding pocket of PDZ1 or PDZ2 (14, 48) were selected indicated for molecular docking. GRK6Act-9 was generated by PyMol (49) with the sequence corresponding to residues 0 to −8 numbered according to the convention as described earlier. The length of the peptide was designed according to our and other studies demonstrating the effect of upstream residues up to −8 position on PDZ-ligand interactions (29, 39, 50). After a short equilibration run (100 ps), a random conformation of GRK6Act-9 was selected for molecular docking. Docking was performed between GRK6Act-9 and PDZ1, PDZ2, or pSer^162^-PDZ2 using ZDOCK (version 3.0.2) (27). Several ZDOCK runs were performed to entertain substantial possible structures. Because the conformational changes between the bound and unbound PDZ1 or PDZ2 is modest (28, 39, 48), it is appropriate to use rigid-body algorithms such as ZDOCK (27). The top ten models from each ZDOCK run were downloaded and viewed by PyMol (49). All models were examined using the following criteria: (i) The C-terminal Leu^0^ of GRK6A (Leu^576^) is inserted in the PDZ domain hydrophobic pocket between the α2 helix and the β2 sheet; (ii) the docked peptide is in the antiparallel orientation to the binding pocket of the PDZ domain; (iii) the extreme C terminus of the docked peptide is not involved in contacts with any part of PDZ domain except the PDZ binding pocket. pSer^162^-PDZ2 was built by replacing the hydrogen atom of the serine residue by a phosphoryl group (-PO_3_^−2^) using the Leap module of AMBER16 (51). The coordinate file for the phosophate group of pSer^162^ was renamed to phosphoserine. The best docking models for PDZ1–GRK6Act-9, PDZ2–GRK6Act-9, and pSer^162^–PDZ2–GRK6Act-9 derived from the above criteria were selected for MD simulations. Files required for MD simulations were prepared by the Leap module of AMBER16 (51). Then, PDZ1/2–GRK6Act-9 complexes were solvated with TIP3P water molecules (52) in a periodically replicated box, neutralized with Na^+^, and energy-minimized over 500 steps including 100 steps of steepest descent minimization using the AMBER16 pmemd module (51).

In addition, the carboxylate-binding site (-GYGF-) in pSer^162^-PDZ2 was replaced by the GAGA sequence, and MD simulations (100 ns) were performed as described for PDZ1/PDZ2–GRK6A. A model established using the Leap module of AMBER16 (51).

### MD simulations

MD simulations were performed using the AMBER16 package with the AMBER force field (ff99SB) and phosaa10 (51) to describe pSer^162^. The simulation details, equilibration, and production simulations, except for the length, were set according to our previous studies (28, 39, 48). In brief, the short runs (0.8 ns) were executed under the NPT ensemble (constant number of particles [N], pressure [P], and temperature [T]) to equilibrate the water molecules. During this equilibration, harmonic restrains were applied to the ligand residues and methodically lowered from *k*_*s*_ = 10 kcal/mol/Å^2^ to 0.1 kcal/mol/Å^2^. Then, equilibration runs (50–70 ns) were continued under the NVT ensemble (constant number of particles [N], volume [V], and temperature [T]) with harmonic restrains *k*_*s*_ = 0.5 kcal/mol/Å^2^ or 0.1 kcal/mol/Å^2^ applied to the ligand. Upon completion of equilibration, production simulations were conducted at 300 K using the canonical NVT ensemble with configurations saved every 2 fs for analysis. Weak harmonic restraints (*k*_*s*_ = 0.1 kcal/mol/Å^2^) were applied to the N-terminal backbone atoms of the ligand and PDZ domain to prevent diffusion from the simulation box. MD simulations were performed for 150 ns each including the equilibration phase. The equilibration phase and production simulations were monitored by computing the RMSD of the Cα atoms from their initial positions (not presented here).

### Data analysis

Results were analyzed using Prism 8.2.1 software (GraphPad). Results represent the mean ± SE of *n* ≥ 3 independent experiments, unless indicated otherwise, and were compared by analysis of variance (ANOVA) with Bonferroni *post hoc* testing. *p* Values <0.05 were considered statistically significant.

## Data availability

Data for all tables are contained within the article. Data for all figures are available from Tatyana Mamonova (tbm7@pitt.edu).

## Supporting information

This article contains supporting information.

## Conflict of interest

The authors declare that they have no conflicts of interest with the contents of this article.

## Footnotes

† Human proteins are indicated by three-letter uppercase convention; genes in italics. Only the first letter is uppercase for the corresponding nonhuman protein or gene.
