# Supplementary material for: Multisite NHERF1 phosphorylation controls GRK6A regulation of hormone-sensitive phosphate transport
Source: J Biol Chem. 2021 Feb 24;296:100473. doi: 10.1016/j.jbc.2021.100473 (PMC8042174; doi:10.1016/j.jbc.2021.100473)
Supplement: Figures S1 to S6 and Table S1 [file mmc1.docx]

**Supporting Information**

**Multisite NHERF1 phosphorylation controls GRK6A regulation of hormone-sensitive phosphate transport**

**Maria Vistrup-Parry^#^, W. Bruce Sneddon^‡^, Sofie Bach^#^, Kristian Strømgaard^#^, Peter A. Friedman^‡^, Tatyana Mamonova^‡^***

**From the ^‡^Laboratory for GPCR Biology, Department of Pharmacology and Chemical Biology,**

**University of Pittsburgh School of Medicine, Pittsburgh, PA, USA; ^#^ Center for Biopharmaceuticals, Department of Drug Design and Pharmacology, University of Copenhagen, Jagtvej 162, DK-2100 Copenhagen, Denmark**

Running title: Phosphoserine 162 of PDZ2 orchestrates binding to GRK6A for PTH-sensitive phosphate transport

Keywords: PDZ domain, parathyroid hormone (PTH), G protein-coupled receptor kinase 6A (GRK6A), phosphate transport, PDZ-ligand interaction, binding affinity, simulation

*Corresponding author: Tatyana Mamonova, University of Pittsburgh School of Medicine, Department of Pharmacology & Chemical Biology, Thomas E. Starzl Biomedical Science Tower, 200 Lothrop Street, Pittsburgh, PA 15261. Email: [tbm7@pitt.edu](mailto:tbm7@pitt.edu)

**FIGURES**

**
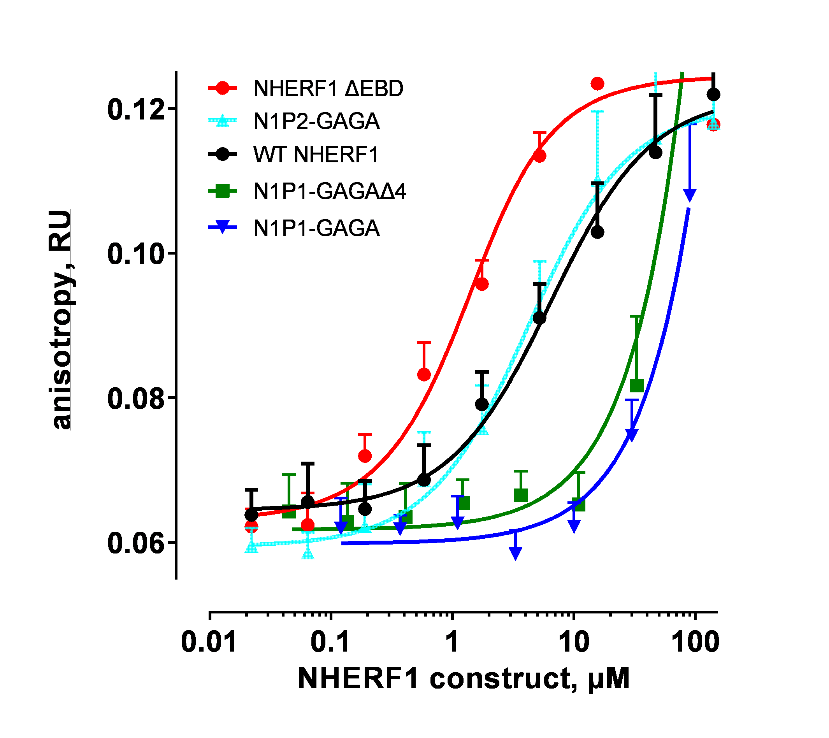
**

**Figure S1. Binding affinities between NHERF1 or NHERF1 constructs and GRK6A**. WT NHERF1 and N1P2-GAGA (PDZ1 intact) interact with GRK6Act-22 with a similar binding affinity, whereas N1P1-GAGA (PDZ2 intact) binds GRK6Act-22 much weaker. Deletion of the C-terminal PDZ-binding motif (-FSNL^358^) of NHERF1 (N1P1-GAGAΔ4) does not affect the binding. The binding affinity between NHERF1 or NHERF1 constructs and the FITC-labeled GRK6Act-22 peptide was analyzed by fluorescent anisotropy binding assay (n=3).

**A**


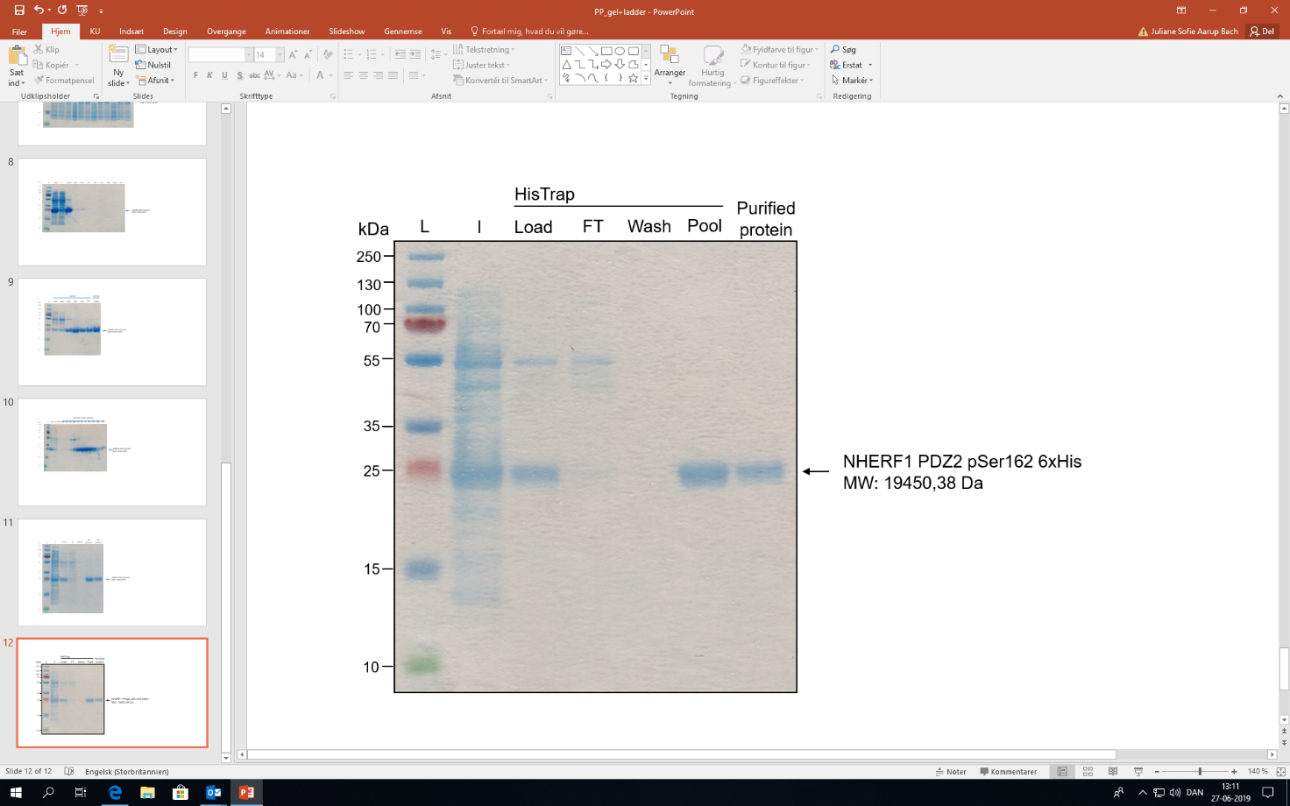


**B**

**C**


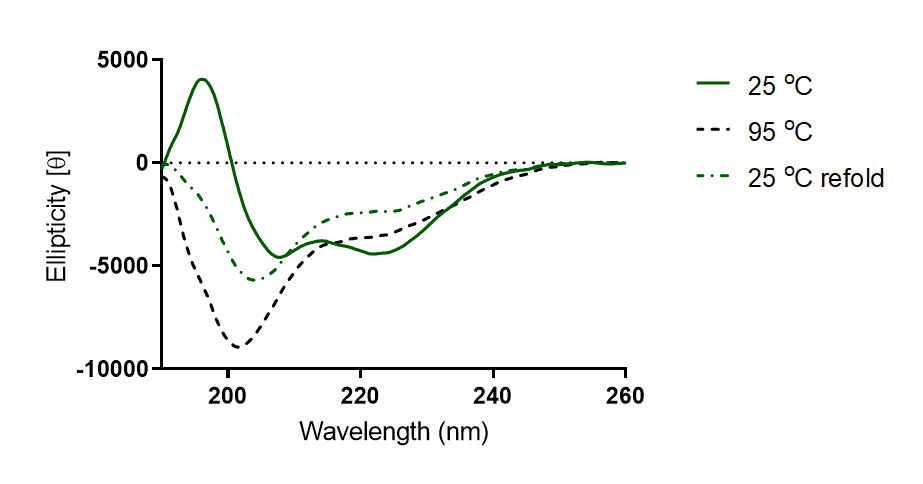


**Figure S2.** **Purification and analysis of pSer^162^-PDZ2** **6xHis**. *A*, Coomassie stained SDS-PAGE of pSer^162^-PDZ2 6xHis showing the induced (I) protein from expression, fractions from the HisTrap purification incl. the column load, flow through (FT), column wash and pooled sample, and the purified protein product; *B,* LC-MS and UPLC chromatograms indicating the molecular weight and purity of pSer^162^-PDZ2 6xHis; *C*, the secondary structure was confirmed by circular dichroism. Three accumulated spectra (n=3) were obtained from 260-190 nm at 25 ⁰C (green line), 95 ⁰C (dotted black line) and refolding at 25 ⁰C directly after the 95 ⁰C scan (dotted green line). Data shown does not exceed a high-tension voltage above 700 V.


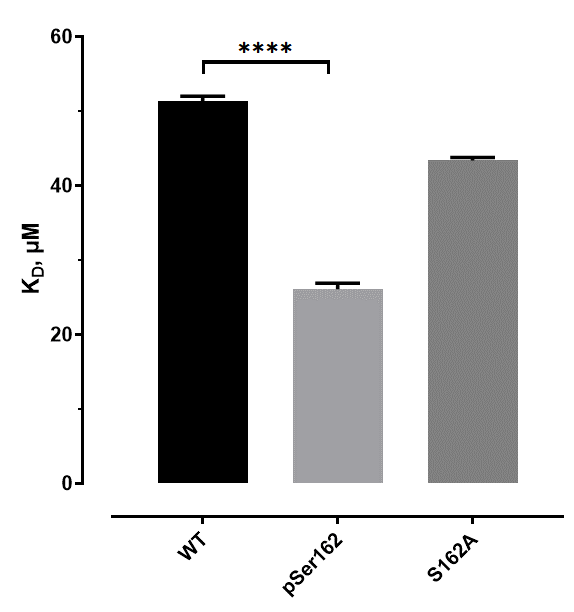


**Figure S3. Binding affinities between PDZ2 mutants and GRK6Act-22.** pSer^162^-PDZ2 interacts with the GRK6Act-22 peptide with 2-fold higher binding affinity compare to WT PDZ2. Results report the mean ± SD (n=5, ****, p < 0.0001, ANOVA).


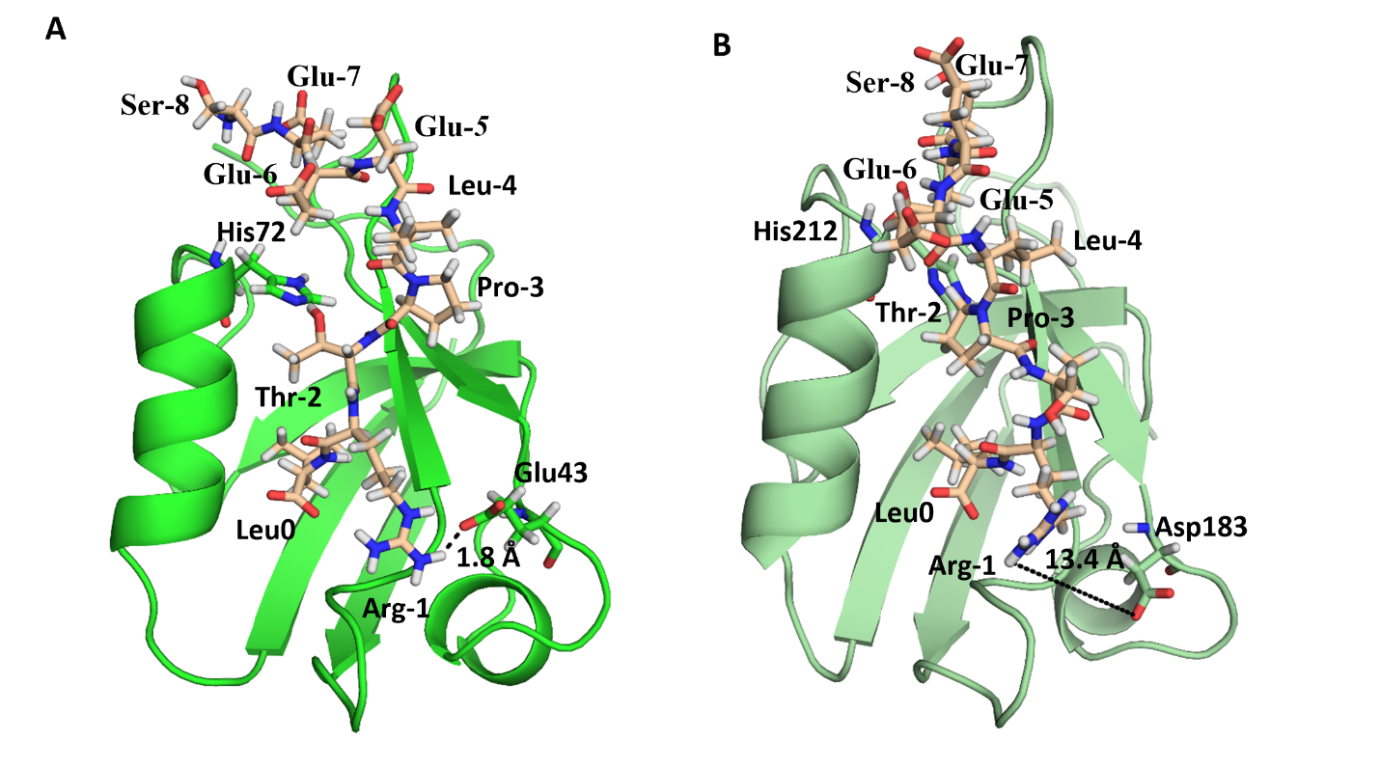


**Figure S4. The representative computational model of NHERF1 PDZ domains in complex with GRK6Act-9.** The PDZ1 and PDZ2 domains are highlighted in green cartoon, whereas the GRK6Act-9 peptide (-SEEELPTRL) is represented in wheat sticks. *A*, the average distance (1.8 Å) between the carboxylate oxygens of Glu^43^ (Oε^1^ or Oε^2^) of PDZ1 and the side chain (NHη^2^ group) of Arg^-1^ of GRK6A permits the formation of strong electrostatic interactions; *B*, in contrast, the distance between the carboxylate oxygens of Asp^183^ (Oδ^1^ or Oδ^2^) of PDZ2 and the NHη^2^ group of Arg^-1^ of GRK6A is not stable and fluctuates from 6.8 Å to 13.4 Å along simulation time. The dotted line represents a salt bridge between a hydrogen atom and acceptor. Hydrogen atoms are white, oxygens are red, and nitrogens are blue.


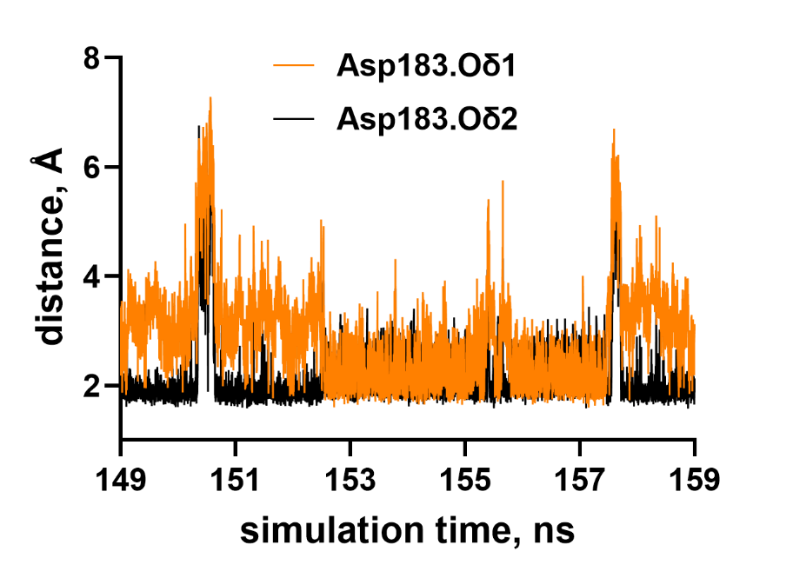


**Figure S5. Distances between** **the carboxylate oxygens of Asp^183^ (Oδ^1^ and Oδ^2^) of pSer^162^-PDZ2 and the NHη^2^ group of Arg^-1^ of GRK6A along the last 10-ns of MD simulation.** The average distance for Oδ^1^-NHη2 (orange) and Oδ^2^-NHη^2^ (black) is 2.9 Å and 2.2 Å, respectively. Plausible electrostatic interactions involved Asp^183^ of pSer^162^-PDZ2 and Arg^-1^ of GRK6A are discussed in the text.


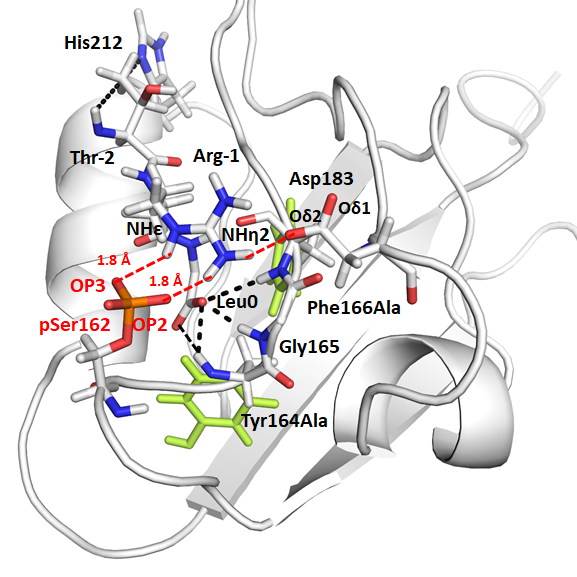


**Figure S6. Computational model of pSer^162^-PDZ2-GAGA bound the GRK6Act-9 peptide.** Substitution of Tyr^164^ (green stick) and Phe^166^ (green stick) from GYGF of pSer^162^-PDZ2 by Ala (white) does not change the plausible electrostatic network involved pSer^162^-Arg^-1^-Asp^183^ shown as red dotted lines. Hydrophobic interactions are disrupted along Tyr^164^Ala and Phe^166^Ala substitutions, but not hydrogen bonds between the backbone amide (NH) of Tyr^164^Ala, Gly^165^ and Phe^166^Ala and carboxylate oxygens of Leu^0^ shown as black dotted lines. Hydrogen atoms are white, oxygens are red, and nitrogens are blue.

Table S1

Binding affinity between isolated PDZ1 domain and GRK6Act-22

| **PDZ1 construct (1-140 aa)** | ***K*_D_, µM** |
| --- | --- |
| GRK6Act-22 | 6.6 ± 0.3^1^ |

^1^ Means and standard deviations (SD) are given (*n=3*).
